# Characterization of Sudan Ebolavirus infection in ferrets

**DOI:** 10.18632/oncotarget.17694

**Published:** 2017-05-08

**Authors:** Andrea Kroeker, Shihua He, Marc-Antoine de La Vega, Gary Wong, Carissa Embury-Hyatt, Xiangguo Qiu

**Affiliations:** ^1^ Special Pathogens Program, National Microbiology Laboratory, Public Health Agency of Canada, Winnipeg, Manitoba, Canada; ^2^ Department of Medical Microbiology, University of Manitoba, Winnipeg, Manitoba, Canada; ^3^ Department of Immunology, University of Manitoba, Winnipeg, Manitoba, Canada; ^4^ Département de microbiologie-infectiologie et d'immunologie, Université Laval, Quebec City, Québec, Canada; ^5^ CAS Key Laboratory of Pathogenic Microbiology and Immunology, Institute of Microbiology, Chinese Academy of Sciences, Beijing, China; ^6^ Canadian Food Inspection Agency, National Centre for Foreign Animal Disease, Winnipeg, Manitoba, Canada

**Keywords:** ferrets, Sudan, Ebolavirus, animal model, characterization

## Abstract

Sudan virus (SUDV) outbreaks in Africa are highly lethal; however, the development and testing of novel antivirals and vaccines for this virus has been limited by a lack of suitable animal models. Non-human primates (NHP) remain the gold standard for modeling filovirus disease, but they are not conducive to screening large numbers of experimental compounds and should only be used to test the most promising candidates. Therefore, other smaller animal models are a valuable asset. We have recently developed a guinea-pig adapted SUDV virus that is lethal in guinea pigs. In our current study, we show that ferrets are susceptible to wild-type SUDV, providing a small animal model to directly study clinical isolates, screen experimental anti-SUDV compounds and potentially study viral transmission.

## INTRODUCTION

The *Filoviridae* family consists of the *Ebolavirus*, *Marburgvirus* and *Cuevavirus* genera. Historically, Ebola virus (EBOV; *Zaire ebolavirus* species) has been the most common and deadly of the filoviruses. Therefore, the research community has largely focused on the development of EBOV animal models, tools, vaccines and therapeutics and has been successful in producing several compounds that have reached the late stages of clinical trials [1] [2]. In light of this success, it is now possible to extend further research towards the discovery of pan-filovirus vaccines and therapeutics. However, animal models that are susceptible to all ebolaviruses species will need to be established first in order to directly evaluate whether pan-filovirus vaccines and therapeutics provide cross-protection.

Nonhuman primates (NHP) most closely mimic clinical manifestations of filovirus infections in humans and have provided invaluable insight into the pathogenesis and course of filovirus disease. Yet due to ethical, cost and space restraints that accompany NHP studies, smaller animal models such as mice or guinea pigs are typically the first choice for initial filovirus drug, vaccine and pathogenesis studies. Although wild-type filoviruses do not cause significant disease in adult, immunocompetent rodents, these viruses have been adapted to rodents so that virulence and lethality are observed [3] [4] [5]. Even though these rodent models do not reproduce all hallmark clinical signs of filovirus disease, their relative ease-of-use and low cost make them attractive first options for evaluating anti-viral prophylactics and therapeutics.

In this study we focused on characterizing ferrets as a novel intermediate animal model for Sudan virus (SUDV; member of the *Sudan ebolavirus* species), for bridging experimental results from rodents to NHPs. SUDV is endemic in South Sudan and the Republic of Uganda and is highly lethal to humans with an average case fatality rate of 53%. The 2000-2001 SUDV outbreak in Uganda was the second largest filovirus outbreak to date, and resulted in 224 deaths in 425 total cases (CFR of 53%) [6]. Unlike EBOV, there is a comparative lack of experimental medical countermeasures against SUDV, and animal models for SUDV are just beginning to emerge. In 2014, it was shown that AG129 (alpha/beta/gamma interferon receptor knockout) mice are susceptible to wild-type SUDV infections [7]. In addition, we recently characterized a guinea pig-adapted variant, which was uniformly lethal to guinea pigs [5].

However, a better strategy would be to screen candidate drugs in immunocompetent small animals using wild-type SUDV to avoid the need to develop rodent-adapted models, before progression to studies in NHPs. Ferrets and pigs initially emerged as excellent models of human respiratory diseases as their lung physiology closely mimics that of humans. Interestingly, among characterization of many respiratory virus infections such as various influenza strains [8], respiratory syncytial virus [9], Nipah virus [10], and coronaviruses [11], other viruses have also recently been tested in ferrets including hepatitis E [12], and three species of ebolavirus [13] [14].

## RESULTS

### Survival, viremia and clinical symptoms in ferrets infected with SUDV

To investigate whether ferrets were susceptible to wild-type SUDV, groups of six animals were inoculated via the intramuscular (IM) or the intranasal (IN) route with SUDV at a back-titered dose of 1260 x TCID_50_. Our stock virus was originally isolated from acute-phase blood of a patient during the 1976 outbreak in Sudan, and then passaged once in guinea pigs, and twice in Vero E6 cells. Its sequence after these passaging steps did not change in comparison to the original patient sample. In addition, the sequence of virus recovered from the ferrets at time of death was not different from the clinical isolate or the input virus, indicating that the virus did not adapt and was, in fact, “wild-type”. On days 0, 2, 4, 6 post-infection as well as on the day of euthanasia (days 7-9), a clinical assessment was performed where blood samples were taken for a complete blood count with differential, a quantification of serum biochemistry parameters, coagulation factors, and viremia.

**Figure 1:**
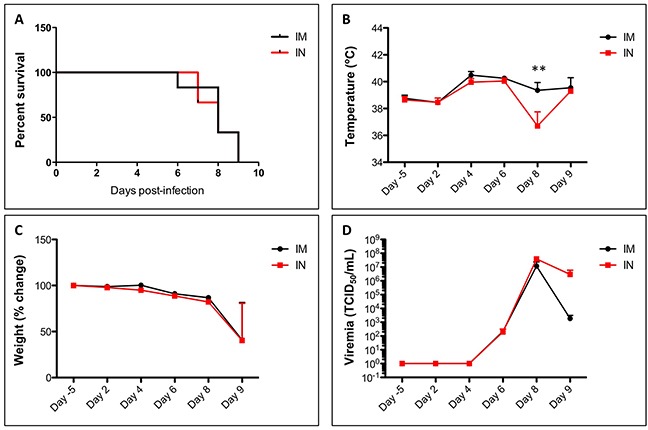
Survival, temperature, weight and viremia in SUDV-infected ferrets Ferrets were infected with SUDV intramuscularly (shown in black) or intranasally (shown in red). All ferrets were monitored for survival **(A)**. In addition, their temperature **(B)** and weight **(C)** were measured daily. Blood samples were taken on days -5, 2, 4, 6, 8 and 9 to assess viremia **(D)** by TCID_50_.

Both IM and IN groups were equally susceptible to infection with wild-type SUDV and succumbed to the disease with a median survival time of 8 days for both groups (Figure 1A). Animals were given a clinical score every day based on visible disease symptoms such as changes in weight and temperature, activity, food and water intake, and respiration, as well as urine and feces. Signs of illness were observed beginning on day 4, and include decreased activity and food and water intake. Respiration rate was increased, and there was a notable absence of urine in severely ill animals. Diarrhea was observed early after the onset of illness but dry/no feces were more prevalent in the animals during advanced SUDV disease. A fever was observed beginning at day 4 post infection (Figure 1B) and a 10-20% decrease in weight between days 6 and 9 (Figure 1C). Viremia was detectable by TCID_50_ by day 6 in both groups (Figure 1D) and by RT-qPCR by day 4 (Supplementary Figure 3A). While similar peak titers were reached by TCID_50_, the intranasal group trended towards slightly higher genome copies than the intramuscular group at later timepoints such as days 7-9; however, this difference did not reach statistical significance.

**Figure 2:**
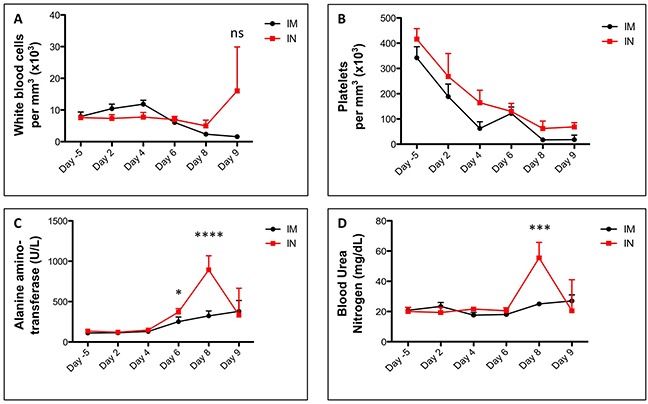
Blood counts and biochemistry in SUDV-infected ferrets Ferrets were infected with SUDV intramuscularly (shown in black) or intranasally (shown in red). White blood cells **(A)** and platelets **(B)** were counted from whole blood using the Abaxis HM5 system. Blood enzyme levels for alanine aminotransferase **(C)** and blood urea nitrogen **(D)** were measured from whole blood using the Abaxis VS2 system. All data points were collected on days -5, 2, 4, 6, 8 and 9.

The course of disease did not substantially affect the absolute count of white blood cells (Figure 2A), lymphocytes (Supplementary Figure 1A), monocytes (Supplementary Figure 1B), neutrophils (Supplementary Figure 1C) or red blood cells (Supplementary Figure 1D) but platelet numbers dropped sharply in both IN and IM groups beginning at days 2-4 post infection (Figure 2B). Other abnormalities in the blood were also apparent by day 4 post-infection. Both IM and IN infection induced a decrease in albumin and calcium levels, an increase in globulin, alkaline phosphatase, alanine aminotransferase, and amylase (Supplementary Figure 2) while maintaining a stable total level of protein. In addition, the IM group saw an increase in sodium levels, and variable patterns of blood urea nitrogen, phosphorus and potassium, while the intranasal group exhibited an increase in blood urea nitrogen, bilirubin, phosphorus and variable levels of glucose and sodium (Supplementary Figure 2). Overall these parameters indicated that by day 4 post infection, multiple organs and the coagulation system were malfunctioning and that the animals were likely reacting to systemic viral infection, inflammation and hemorrhage.

**Figure 3:**
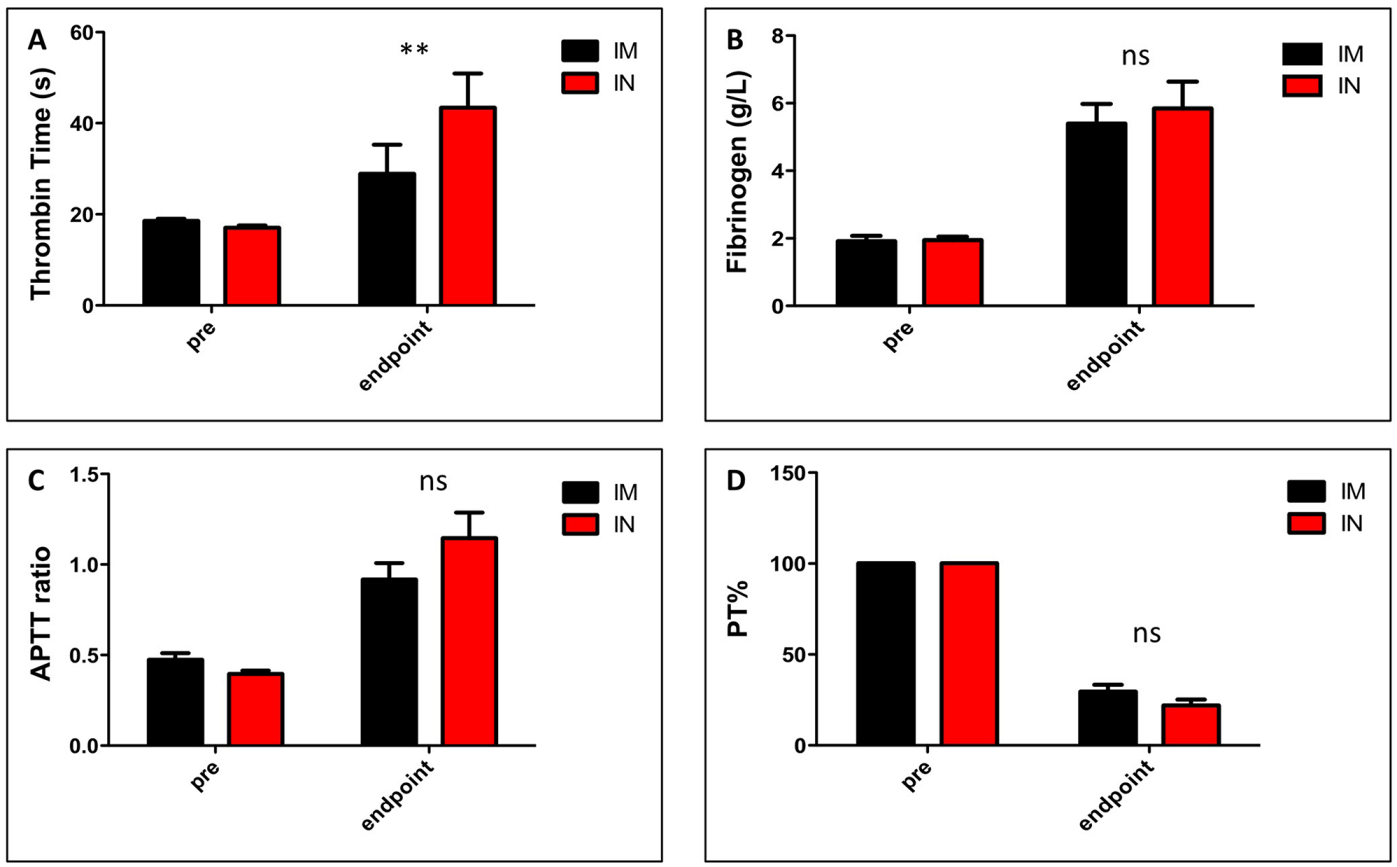
Markers of coagulation in SUDV-infected ferrets Ferrets were infected with SUDV intramuscularly (shown in black) or intranasally (shown in red). Markers of coagulation in the blood were analyzed using the STart4 analyzer. For each ferret, blood samples were taken pre-infection and also at time of death (days 7-9). Plasma samples were mixed with a known amount of thrombin and the time until clotted was measured **(A)**. A normal value = 21s while higher values indicate the presence of coagulation factors. Plasma clotting times were assayed and fibrinogen levels were calculated based on a standard curve **(B)**. Activated partial thromboplastin times **(C)** and % partial thromboplastin times **(D)** were measured from plasma before infection and at time of death.

We also measured the presence of coagulation factors in the blood. The presence of thrombin and fibrinogen in the blood is generally indicative of coagulation and results from the activity of either an intrinsic or extrinsic coagulation pathway. To assay for thrombin, plasma samples from each ferret were spiked with a known amount of thrombin that activated blood clotting in about 21s in control (pre-infection) samples; faster clotting times in endpoint samples indicated that additional thrombin was present (Figure 3A). In order to measure fibrinogen levels, a standard curve was produced using control plasma samples containing serial dilutions of known amounts of fibrinogen; the clotting time was then measured in ferret samples and fibrinogen levels were calculated based on the standard curve (Figure 3B). All ferrets had 2-4 fold higher levels of fibrinogen at time of death compared to pre-infection. The measurement of activated partial thromboplastin time (APTT) determined the activity of the intrinsic coagulation pathway (factors XII, XI, IX, VIII, X, V, II and I) (Figure 3C); the ratio displayed in our results indicates that the activity in the ferret plasma samples was elevated compared to the manufacturer's control plasma sample. Prothrombin time % (PT%) indicates the percentage of normal activity in our ferret samples compared to a manufacturer's control plasma sample and correlates with the involvement of the extrinsic coagulation pathway (factors II, V, VII, and X); this value was decreased in both IN and IM groups at time of death (Figure 3D).

### Virus shedding and spread to internal organs of ferrets infected with SUDV

To investigate viral shedding after SUDV infection, oral and rectal swabs, as well as nasal washes collected from the animals were used to determine virus at various time points by RT-qPCR and TCID_50_. Virus was detected earlier and was generally higher in ferrets that received IN inoculation compared to the IM infection, including nasal washes (IM: 10^1^-10^3^ GEQ/ml and 10^2^-10^4^ TCID_50_/ml; IN: 10^2^-10^4^ GEQ/ml and 10^2^-10^9^ TCID_50_/ml) (Supplementary Figures 3B, 4A) and oral swabs (IM: 10^1^-10^4^ GEQ/ml and 10^2^-10^4^ TCID_50_/ml; IN: 10^2^-10^5^ GEQ/ml and 10^2^-10^6^ TCID_50_/ml) (Supplementary Figures 3C, 4B). In contrast, rectal shedding was only detected in 1 or 2 animals per group (10^1^-10^3^ GEQ/ml and 10^3^-10^4^ TCID_50_/ml) (Supplementary Figures 3D, 4C).

**Figure 4:**
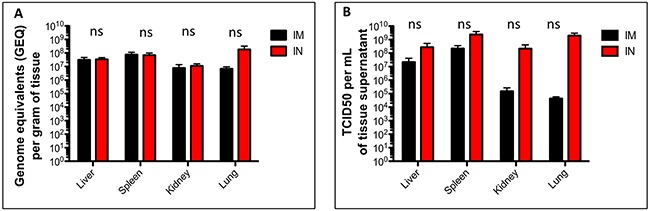
Viral burden in tissues of SUDV-infected ferrets Ferrets were infected with SUDV intramuscularly (shown in black) or intranasally (shown in red). At time of death (days 7-9) the liver, spleen, kidney, heart, lung and pancreas were collected and assessed for the presence of viral RNA by **(A)** qRT-PCR and **(B)** infectious virus by TCID_50_.

In order to assess systemic viral spread to different organs, liver, spleen, kidneys, heart, and lungs were harvested at time of death and assessed for viral titers. In both IM and IN groups, SUDV had spread systemically and had infected the majority of internal organs in each animal. Most organs in both IM and IN groups consistently reached a titer of ∼10^7^ -10^8^ GEQ/g tissue, although the average GEQs in the IN group were slightly higher (Figure 4A). Similarly, most tissues contained infectious virus as measured by TCID_50_ and titers in the IN group were generally higher than in the IM group, particularly in the lungs (Figure 4B).

### Histopathology, and immunohistochemistry findings in the internal organs of ferrets infected with SUDV

**Figure 5:**
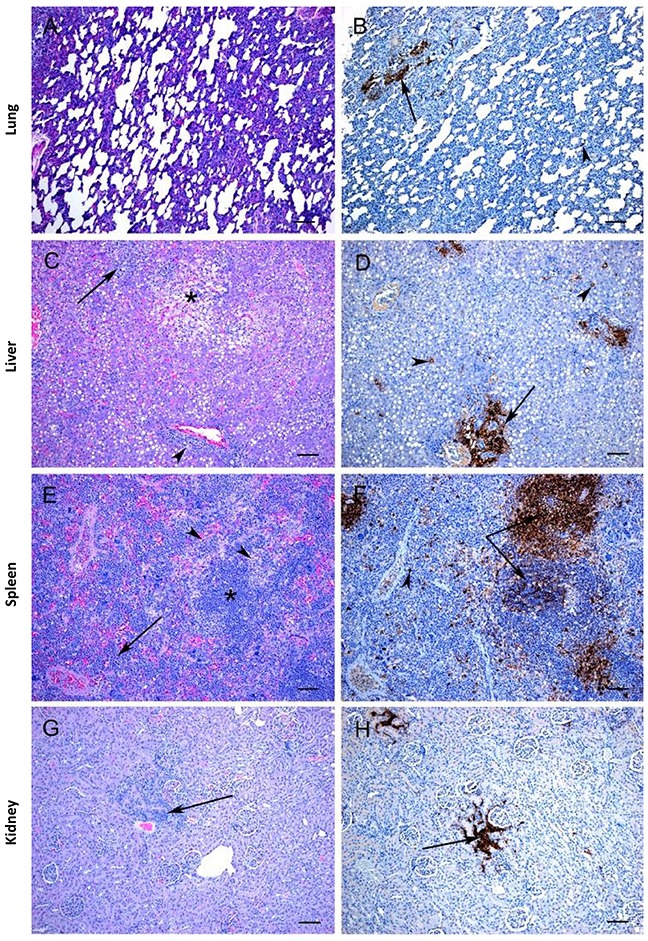
Histopathology and immunohistochemistry findings in ferrets inoculated with SUDV via the IM route **(A)** Mild lung changes are characterized by increased cellularity of the alveolar walls. **(B)** Viral antigen is detected in scattered cells with the morphology of macrophages (arrowhead) as well as occasionally in perivascular areas (arrow). **(C)** There are scattered areas of severe vacuolar degeneration and loss of hepatocytes (*), foci of inflammation and necrosis (arrow) and periportal infiltration of inflammatory cells (arrowhead). **(D)** Viral antigen is observed with individual hepatocytes (arrowheads) and in periportal areas (arrow). **(E)** There is hypercellularity of the splenic cords (arrow) and scattered lymphocytolysis (arrowhead, *=periarterial lymphatic sheath). **(F)** Viral antigen is observed in scattered macrophages throughout the red pulp (arrowhead) and within periarterial lymphatic sheath areas (arrows). **(G)** Foci of interstitial nephritis (arrow) are occasionally observed. **(H)** Viral antigen is detected in the areas of interstitial nephritis (arrow). All tissues were collected at time of death (days 7-9). **A,C,E,G**: H&E stain. Bar = 100 μm.

**Figure 6:**
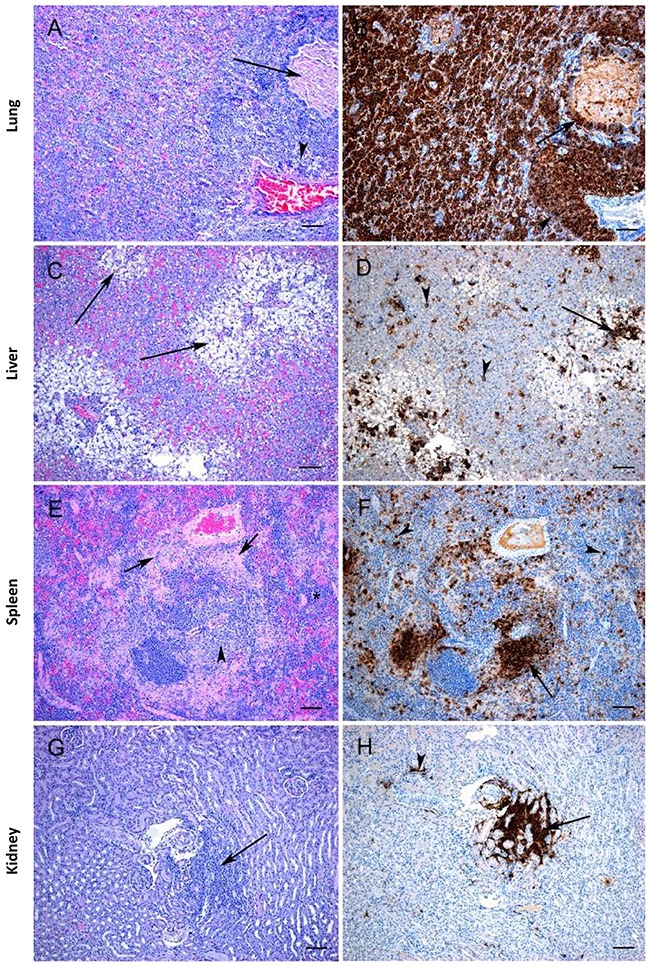
Histopathology and immunohistochemistry findings in ferrets inoculated with SUDV via the IN route **(A)** Severe bronchointerstitial pneumonia with thickened alveolar walls (edema, type II pneumocyte hyperplasia) and filling of alveolar spaces by neutrophils and macrophages leading to loss of air spaces. Bronchiolitis (arrow) and perivascular inflammation (arrowhead) is also observed. **(B)** Immunopositive staining for viral antigen is widespread affecting many components including the inflammatory infiltrate within the alveolar spaces as well as bronchiolar epithelial cells (arrow). **(C)** There are numerous areas of severe vacuolar degeneration and loss of hepatocytes (arrows). **(D)** Viral antigen is detected with individual hepatocytes (arrowheads) and associated with areas of degeneration (arrow). **(E)** There is hypercellularity of the splenic cords (*), scattered lymphocytolysis (arrowhead), and areas of necrosis (arrows). **(F)** Viral antigen is observed in scattered macrophages throughout the red pulp (arrowheads) and within periarterial lymphatic sheath areas (arrow). **(G)** Foci of interstitial nephritis (arrow) are occasionally observed. **(H)** Viral antigen is detected in the areas of interstitial nephritis (arrow). All tissues were collected at time of death (days 7-9). **A,C,E,G**: H&E stain, Bar = 100 μm.

In both the IN and the IM inoculated groups there were several histopathologic changes observed in the tissues, which were associated with the presence of SUDV antigen. In the lungs of the IM inoculated animals, lesions were very mild and were characterized by a slight increase in inflammatory cells within the alveolar walls (Figure 5A) and antigen was only occasionally detected, primarily within macrophage-like cells and in perivascular areas (Figure 5B). In contrast, the lungs of the IN inoculated animals showed multifocal to diffuse severe bronchointerstitial pneumonia (Figure 6A) and abundant positive immunostaining (Figure 6B). In the livers of both groups there was moderate to severe pathology with diffuse vacuolar degeneration and loss of hepatocytes leading to disruption of normal architecture as well as infiltration of inflammatory cells into the portal areas (Figures 5C, 6C). In both groups viral antigen could be detected within hepatocytes and in periportal areas (Figures 5D, 6D); however, viral antigen was more abundant in the IN-inoculated group. In the spleen of the IM group the only significant lesion was increased cellularity of the splenic cords; however, in one animal there was some lymphocytolysis (Figure 5E). In the IN group, similar lesions were observed; however, in addition there were multifocal areas of necrosis (Figure 6E). Abundant viral antigen was observed within macrophage-like cells throughout the red pulp areas and multifocally within germinal centres (Figures 5F, 6F); however, viral antigen was more abundant in the IN group. Few lesions were observed within the kidneys; however, in both the IN and IM groups there were occasional foci of interstitial nephritis (Figures 5G, 6G), which were associated with the presence of viral antigen (Figures 5H, 6H). No significant lesions were observed in the heart of either group; however, small amounts of viral antigen were detected within capillaries and occasionally within a cardiomyocyte or in the endocardium.

## DISCUSSION

Choosing and developing animal models that closely mimic human physiology and course of disease are important steps towards the development of effective prophylactic and therapeutic options. Although rodent and other small animal models are suitable for large antiviral screens, animal models closely resembling hallmarks of human disease are imperative for follow-up studies to confirm the believability and reliability of pre-clinical results, before testing in humans. Our study demonstrates that both IM and IN routes of SUDV inoculation lead to viremia, systemic organ infection and dysfunction, viral shedding, and a similar time to death. This is evidenced by the following indicators: 1) increase in viremia and organ viral loads, which is indicative of uncontrolled virus replication and spread; 2) decrease in WBC and LYM counts, which is indicative of compromised immunity; and 3) increases in fibrinogen, APTT, TT, and decreases in PLT and PT%, which is indicative of disseminated intravascular coagulopathy. While the overall kinetics of the viral infection was similar between the two groups, viral loads were consistently higher in the IN infected animals and appeared earlier in the nasal and oral swabs as well as the lung tissue.

SUDV infection in ferrets shares many similarities with SUDV disease in other models, such as IFNα/βR^−/−^ knockout mice, guinea pigs and NHPs in addition to humans (Table 1). Fever, weight loss, leukopenia, decreases in PLT counts but no external haemorrhaging was observed in all animal models; however, haemorrhage can be observed in human patients. Rash was not observed in our animals; however, they were documented in another SUDV ferret study [13] and is sometimes observed in NHPs, and can be seen in humans. Coagulopathy was observed in most animal models (knockout mice, guinea pigs, ferrets) in addition to humans. Increases to ALP, ALT, AST, BUN, and decreases to CA and TP levels were observed in ferrets and NHPs. Dysregulated cytokines, such as increased levels of TNF-α and nitric oxide, was observed in ferrets and humans (Table 1).

**Table 1:** Summary of SUDV disease in mice, guinea pigs, ferrets, NHPs and humans

References		Knockout mice (wild-type)	Guinea pigs (GPA)	Ferrets (wild-type)	Ferrets (wild-type)	NHPs (wild-type)	Humans (wild-type)
		[16]	[5]	Current study	[13]	[17]	[18] [19]
Fever		Yes	Yes	Yes	Yes	Yes	Yes
Weight loss / weakness		Yes	Yes	Yes	Yes	Yes	Yes
Leukopenia		Yes	Yes	Yes	Yes	Yes	Yes
Platelet count		Decrease	Decrease	Decrease	Decrease	Decrease	Decrease
Dyspnea / hypoxemia		N/A	N/A	N/A	N/A	Yes	Yes
Heart rate		N/A	N/A	N/A	N/A	Increase	
Rash		N/A	No	No	Yes	Sometimes	Yes
Gastrointestinal symptoms		N/A	N/A	N/A	N/A	Yes	Yes
Neurologic complications		N/A	N/A	N/A	N/A	N/A	Yes
External hemorrhage		No	No	No	No	No	Yes
Coagulopathy		Yes	Yes	Yes	Yes	N/A	Yes
Changes in serum levels	Albumin	N/A	Decrease	Decrease	Decrease	Decrease	
	Alkaline phosphatase	N/A	Increase	Increase	Increase	Increase / No change	
	Alanine transaminase	Increase	No change	Increase	N/A	Increase	
	Aspartate transaminase	Increase	N/A	Increase	Increase	Increase	
	Amylase	no change	N/A	Increase	Increase	Decrease	
	Total bilirubin	N/A	N/A	Increase / no change	N/A	Increase / no change	
	Blood urea nitrogen	no change	Increase	Increase / no change	Increase	Increase	
	Creatinine	N/A	no change	no change	Increase	Increase / no change	
	GGT	N/A	N/A	N/A	Increase	Increase / no change	
	Calcium	N/A	Decrease	Decrease	N/A	Decrease	
	Glucose	N/A	N/A	Increase	N/A	N/A	
	Sodium	N/A	N/A	Decrease / no change	N/A	N/A	
	Potassium	N/A	N/A	Increase / no change	N/A	N/A	
	Phosphorus	N/A	no change	Increase / no change	N/A	N/A	
	Total protein	N/A	Decrease	Decrease	no change	Decrease / no change	
Dysregulated cytokines	TNF-alpha	N/A	N/A	N/A	Increase	N/A	Yes
	Nitric oxide	N/A	N/A	N/A	Increase	N/A	

Logistically, ferrets are more demanding in terms of housing space, maintenance and cost compared to rodents. In addition, there are few immunological reagents available to study T-cell responses in ferrets although several were previously developed by Pillet et al [15]. Alternatively, some human reagents have been used successfully in other studies and could be validated for this purpose in the future. However, the ferret model also provides several major advantages. Of particular value is the ability to use clinical isolates without the need for prior adaptation [8] [13] [14]. All three ebolaviruses tested in another study [13] and the SUDV species used in our current study were highly infectious and uniformly lethal, which has not been previously possible without adaptation except in non-human primates. Our study also adds several valuable and unique aspects to the characterization of SUDV-infected ferrets. For example, we compared two different common routes of infection. IN represents a mucosal infection, which is thought to be the most common natural infection route among people, whereas IM inoculation is similar to a needle-stick injury in the lab. Interestingly, our data demonstrated that the rate and spread of virus is very similar in both routes of infection with the exception that nasal, oral and lung tissues replicated the virus to approximately 10-fold higher titers. We also included novel data regarding viral shedding from mucosal tissues that demonstrated that ferrets may hold promise for future transmission studies, which currently rely solely on the use of nonhuman primates. Although we did not directly test transmission in this study, we observed high levels of oral and nasal viral shedding suggesting that ferrets may readily transmit to other ferrets via these routes. The ability to study ebolavirus transmission without the use of nonhuman primates would be highly beneficial both in terms of cost, feasibility and ethics approval.

Overall, the development of a ferret model for ebolaviruses provides a useful intermediate animal model that may be able to bridge rodent and nonhuman primate studies, as well as provide a new means of studying viral transmission and testing vaccines and therapeutics.

## MATERIALS AND METHODS

### Ethics statement

The animal work for this study was performed in the biosafety level-4 (BSL-4) facility at the Canadian Science Centre for Human and Animal Health (CSCHAH) in Winnipeg, Canada. All experiments were approved by the Animal Care Committee of the CSCHAH, in accordance with guidelines from the Canadian Council on Animal Care. As per protocol, animals were acclimatized for 7 days prior to infection, were given food and water *ad libitum* and were monitored twice daily. Environmental enrichment was also provided in the cages throughout the duration of the study.

### Animals and viruses

Twelve six-month old female ferrets (*Mustela putorius furo*) were purchased from Marshall BioResources (New York, USA) and received anti-coccidials and vaccinations against distemper virus and rabies virus prior to commencement of the experiment. The ferrets were randomly assigned into two groups and infected either intramuscularly (IM) or intranasally (IN) using a targeted challenge dose of 1000 x TCID_50_ of SUDV (isolate Boneface). The virus stock we used was originally isolated from the acute-phase blood of a patient during the 1976 outbreak in Sudan, which was passaged once in guinea pigs and twice on VeroE6 cells (GenBank accession no. FJ968794.1). The backtitration indicated that each animal received 1260 x TCID_50_. After infection, all animals were monitored daily for signs of disease and blood was drawn every second day to determine viral load and to evaluate biochemical markers.

### Serum biochemistry, blood counts, and coagulation

Serum biochemistry was evaluated on days 0, 2, 4, 6, and 8 post-infection with the VetScan VS2 blood analyzer (Abaxis, USA) using heparinized blood. To investigate the complete blood cell counts, whole blood was analyzed using a VetScan HM5 hematology system (Abaxis, USA). To investigate coagulation, citrated plasma was used to determine the content of fibrinogen, the amount of activated partial thromboplastin (APTT) generation and percent of partial thromboplastin using a StAart4 instrument (Diagnostica Stago). All assays were run as per manufacturer's instructions.

### Quantification of viral loads by qRT-PCR

Total RNA was extracted from blood, as well as oral, rectal, and nasal swabs, using the QIAamp viral RNA minikit (Qiagen). Total RNA was extracted from organ biopsies using the RNeasy Kit according to the manufacturer's instructions (Qiagen). Reverse transcription-quantitative PCR (Roche Lightcycler 480 RNA Master Hydrolysis Probes kit) was used to determine viral titers with the following para-meters: primers and probes to the L gene: SUDV-L-forward (5′-CAGAAGACAATGCAGCCAGA-3′), SUDV-L-reverse (5′-TTGAGGAATATCCCAC-AGGC-3′), SUDV-L-probeFAM (5′-FAMCTGCTAGCTTGGCCAAAGTCACAAG -BHQ1-3′); and the PCR program: 63°C for 3 minutes, 95°C for 30 seconds, and cycling of 95°C for 15 seconds, 60°C for 30 seconds for 45 cycles.

### Quantification of viral loads by TCID_50_

CV-1 cells were grown to 95% confluence and infected with 10-fold serial dilutions of whole blood for 1 h at 37°C. The inoculum was then removed, and cells were overlaid with fresh MEM plus 2% FBS. At 14 days postinfection, the plates were assessed for the lowest dilution at which 50% of the wells exhibited cytopathology. TCID_50_ per milliliter was calculated according to the Reed-Muench method.

### Data analysis

Data was analyzed using GraphPad Prism software. Survival was compared using the log rank test. A p-value of < 0.05 = *, p-value of <0.01 = **, p-value of <0.001 = ***, p-value of <0.0001 = ****, and ns = not significant.

### Histopathology and immunohistochemistry

Tissues were fixed in 10% neutral phosphate buffered formalin, routinely processed, sectioned at 5 μm and stained with hematoxylin and eosin (HE) for histopathologic examination. For immunohistochemistry (IHC), paraffin tissue sections were quenched for 10 minutes in aqueous 3% hydrogen peroxide. Epitopes were retrieved using Dako Target Retrieval solution (Dako, USA) in a Biocare Medical Decloaking Chamber. The primary antibody applied to the sections was a mouse monoclonal anti-Ebola Sudan (F344G5). It was used at a 1:800 dilution for thirty minutes. They were then visualized using a horse radish peroxidase labelled polymer, Envision® + system (anti-mouse) (Dako, USA) and reacted with the chromogen diaminobenzidine (DAB). The sections were then counter stained with Gill's hematoxylin.

## SUPPLEMENTARY FIGURES



## Abbreviations

CFR: case fatality rate
GEQ: genome equivalents
IM: intramuscular
IN: intranasal
NHP: nonhuman primate
SUDV: Sudan virus
TCID: tissue culture infectious dose
