# Supplementary material for: Characterization of Sudan Ebolavirus infection in ferrets
Source: Oncotarget. 2017 May 8;8(28):46262–72. doi: 10.18632/oncotarget.17694 (PMC5542265; doi:10.18632/oncotarget.17694)
Supplement: Supplementary file 1 [file oncotarget-08-46262-s001.pdf]

Characterization of Sudan Ebolavirus infection in ferrets

SUPPLEMENTARY MATERIALS

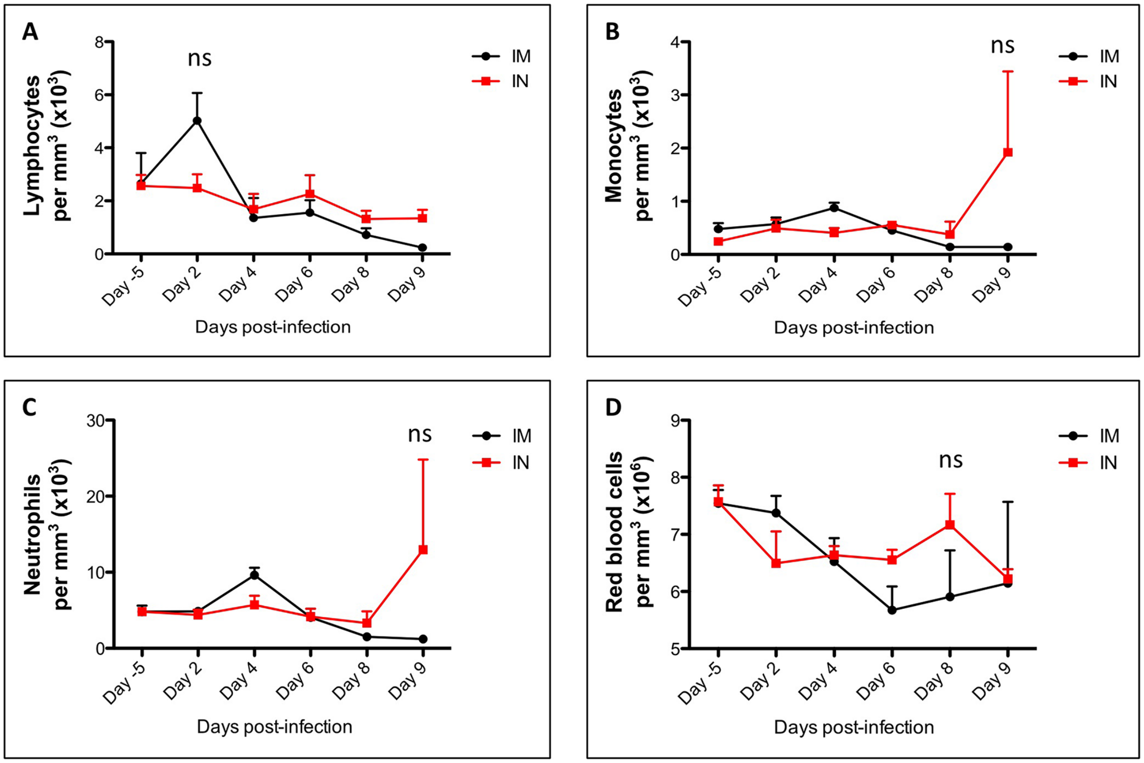

Supplementary Figure 1: Blood counts in SUDV-infected ferrets. Ferrets were infected with SUDV intramuscularly (shown in black) or intranasally (shown in red). On days -5, 2, 4, 6, 8 and 9, whole blood samples were collected and analyzed for lymphocytes **(A)**, monocytes **(B)**, neutrophils **(C)** and red blood cells **(D)** using the Abaxis HM5 system.

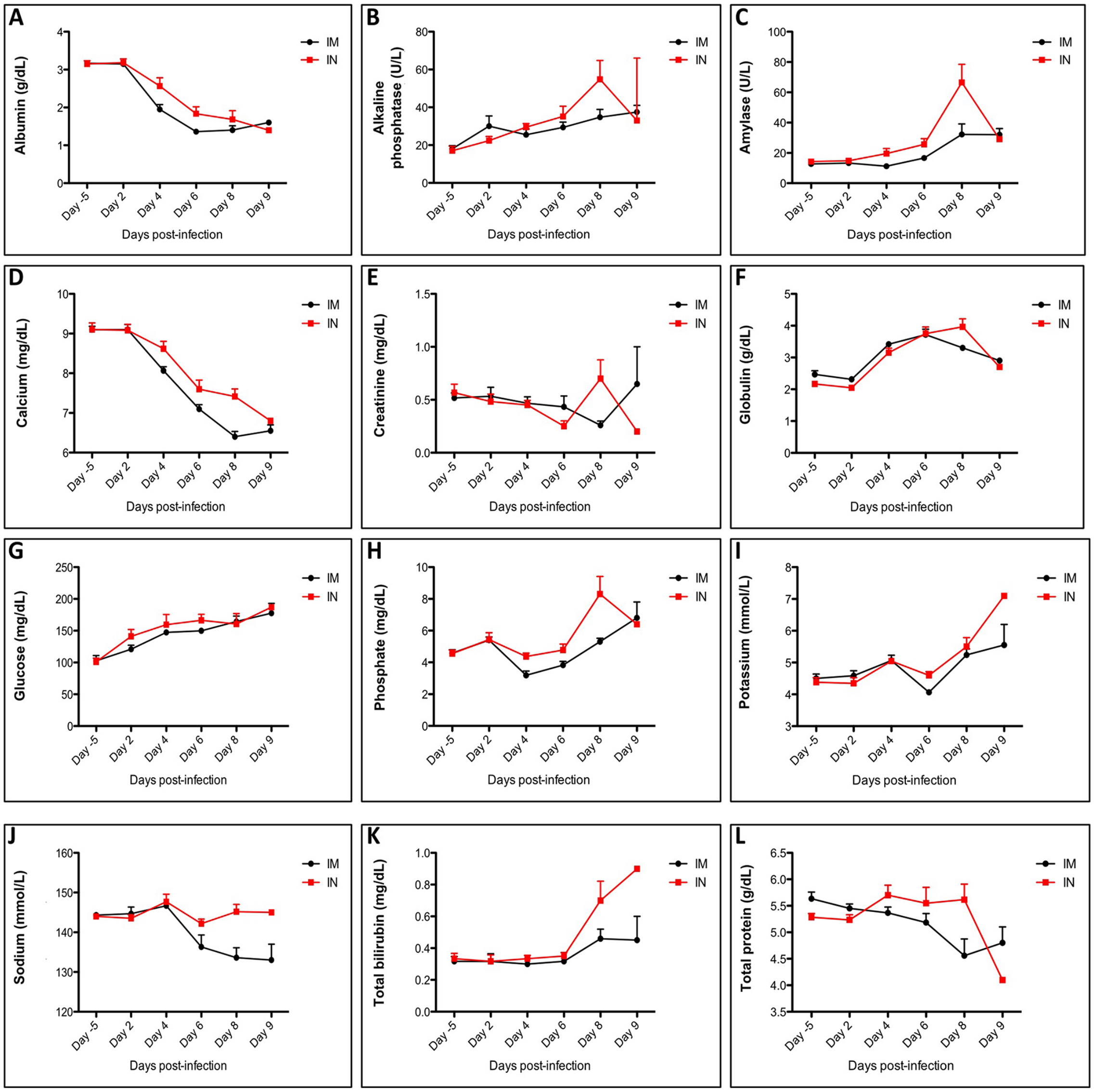

Supplementary Figure 2: Serum biochemistry for SUDV-infected ferrets. Ferrets were infected with SUDV intramuscularly (shown in black) or intranasally (shown in red). On days -5, 2, 4, 6, 8, and 9, whole blood was collected used to measure a panel of proteins and metabolites **(A-L)** using the Abaxis VS2 system.

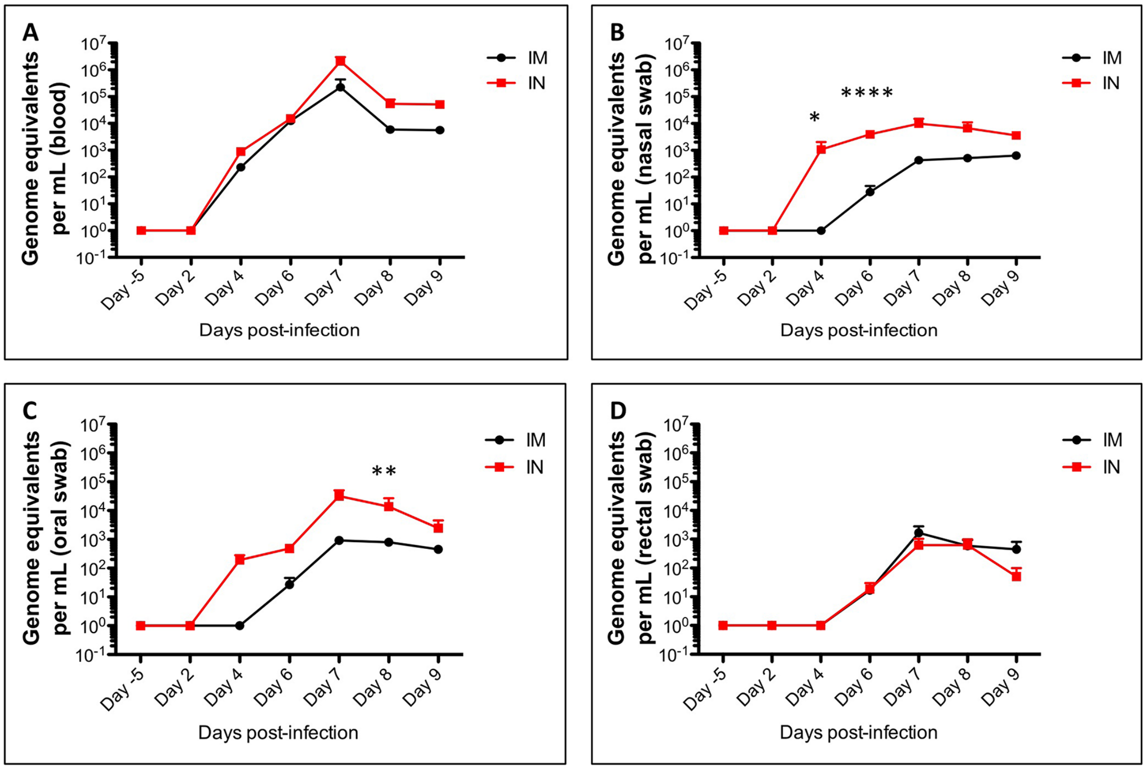

Supplementary Figure 3: Viremia and mucosal swabs from SUDV-infected ferrets. Ferrets were infected with SUDV intramuscularly (shown in black) or intranasally (shown in red). On days -5, 2, 4, 6, 7, 8, and 9, viral RNA was isolated and quantified from the blood **(A)** and from mucosal swabs of the nose **(B)**, mouth **(C)** and rectum **(D)** of infected ferrets.

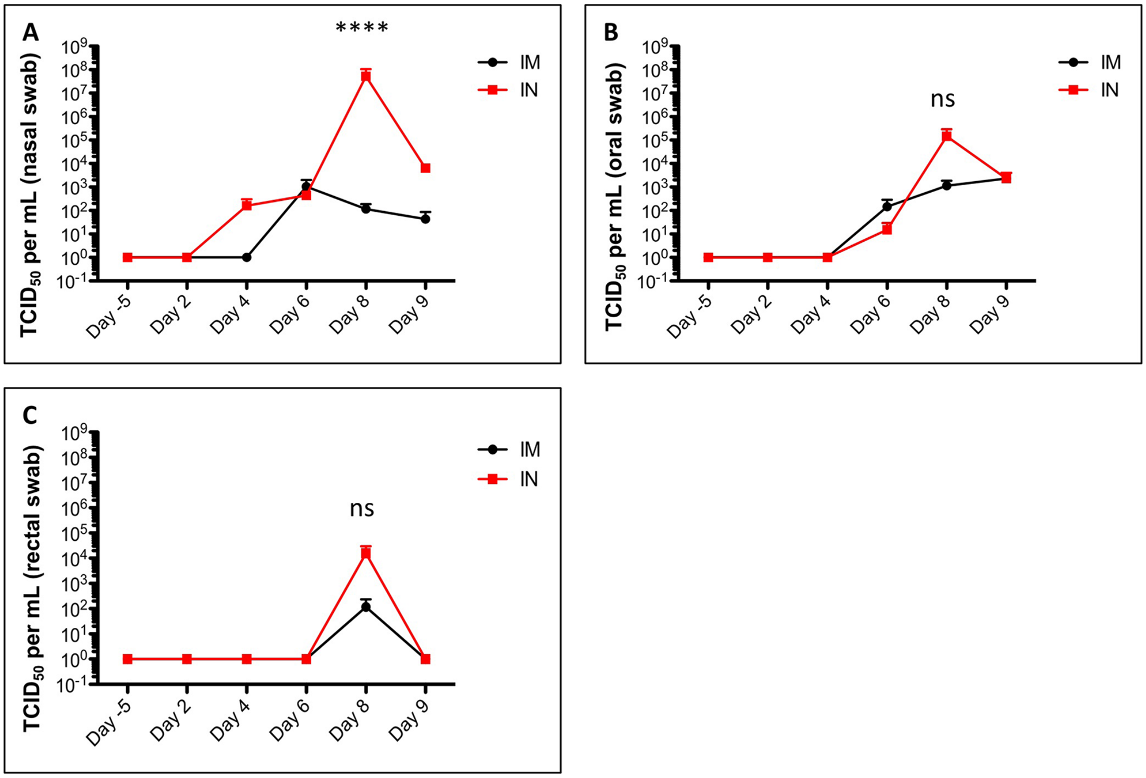

Supplementary Figure 4: Mucosal swabs from SUDV-infected ferrets. Ferrets were infected with SUDV intramuscularly (shown in black) or intranasally (shown in red). On days -5, 2, 4, 6, 8, and 9, infectious virus was isolated from mucosal swabs of the nose **(A)**, mouth **(B)** and rectum **(C)** of infected ferrets and quantified by $TCID_{50}$.
